# Correction to “Losartan Enhances Radiosensitivity by Reversing Immunosuppressive Tumor Microenvironment Induced by Radiotherapy in TNBC”

**DOI:** 10.1111/cas.70528

**Published:** 2026-09-05

X. Wang , C. Liu , Z. Xia , et al., “Losartan Enhances Radiosensitivity by Reversing Immunosuppressive Tumor Microenvironment Induced by Radiotherapy in TNBC,” Cancer Science 117, no. 7 (2026): 1795–1812, 10.1111/cas.70407.42095675 PMC13327069

In the version of this article initially published, the author list and affiliations are incorrect. Cuiwei Liu should be added as the second author in the author list. The updated author list and affiliation are shown below and they have been updated in the online article.

Xu Wang^1,2^ | Cuiwei Liu^1,3,4,5^ | Zihan Xia^1,6,7^ | Qi Chen^1,3,4,5^ | Ting Hu^1,3,4,5^ | Yuting Li^1,3,4,5^ | Dan Han^1,3,4,5^ | Yunjie Song^8^ | Yuxi Ma^1,3,4,5^ | Yanxia Zhao^1,3,4,5^

^1^Cancer Center, Union Hospital, Tongji Medical College, Huazhong University of Science and Technology, Wuhan, China | ^2^The Affiliated Cancer Hospital of Zhengzhou University & Henan Cancer Hospital, Zhengzhou, China | ^3^Institute of Radiation Oncology, Union Hospital, Tongji Medical College, Huazhong University of Science and Technology, Wuhan, China | ^4^Hubei Key Laboratory of Precision Radiation Oncology, Union Hospital, Tongji Medical College, Huazhong University of Science and Technology, Wuhan, China | ^5^Key Laboratory of Biological Targeted Therapy, Huazhong University of Science and Technology, Ministry of Education, Wuhan, Hubei, China | ^6^Laboratory of Experimental Cancer Research, Ghent University, Ghent, Belgium | ^7^Cancer Research Institute Ghent (CRIG), Ghent, Belgium | ^8^State Key Laboratory of Neurology and Oncology Drug Development, Nanjing, China

Xu Wang, Cuiwei Liu, and Zihan Xia are the co‐first authors of this work.

We apologize for this error.

